# Sustainable Intumescent Flame-Retardant Coating with Sericin, Phosphorus, and Silicon for Polyester Fabrics

**DOI:** 10.3390/polym18060682

**Published:** 2026-03-11

**Authors:** Thitirat Inprasit, Dujdow Niyomdacha, Chayutima Promchantuek, Thitima Thangtong, Chutima Vanichvattanadecha, Penwisa Pisitsak

**Affiliations:** 1 Department of Materials and Textile Technology, Faculty of Science and Technology, Thammasat University, Pathum Thani 12121, Thailand; 2 Advanced Composite and Nanotextiles Research Team, National Nanotechnology Center, National Science and Technology Development Agency, Pathum Thani 12120, Thailand; 3 Center of Excellence in Smart Materials, Energy, Biochemistry, Food Technology, and Textile Innovation for Sustainable Environment, Thammasat University, Pathum Thani 12121, Thailand

**Keywords:** aluminum diethylphosphinate, intumescent flame-retardant coating, P/N/Si synergistic system, polyester fabric, sericin, sustainable textile finishing

## Abstract

In this study, we developed an eco-friendly intumescent flame-retardant coating for polyester (PET) fabrics. The coating was formulated with aluminum diethylphosphinate-based flame retardant (P-FR), trisilanol isobutyl-POSS (Si-FR), sericin (SC), and poly(vinyl alcohol) (PVA), using citric acid (CA) as a chemical crosslinker. The coatings were applied to alkaline-treated PET fabrics via the knife-coating technique, followed by drying and curing. P-FR acted as the primary flame-retardant component, while SC and Si-FR served as N/Si synergistic agents that enhanced the performance of P-FR, as demonstrated by an improvement in the UL 94 rating from V-1 to V-0. Thermogravimetric analysis indicated that SC and Si-FR improved the oxidative stability of the char. Flame-retardant finishing increased the limiting oxygen index (LOI) from 21.1% for untreated fabric to 31.7% for treated fabric, while tensile strength increased and elongation at break decreased. Notably, after 50 washing cycles, the treated fabrics retained self-extinguishing behavior, although the UL 94 classification decreased to V-2. Overall, this halogen-free coating system effectively enhanced the flame retardancy of PET fabrics while using environmentally friendly components, indicating its potential for sustainable flame-retardant textile applications.

## 1. Introduction

Polyester fibers are widely used in applications such as clothing (e.g., sportswear, casual wear), home furnishings (e.g., carpets, upholstery), and industrial products (e.g., ropes, tire cords, and filters). Among synthetic fibers, poly(ethylene terephthalate) (PET) dominates the market due to its strength, processability, recyclability, and cost-effectiveness [1,2]. However, its inherent flammability poses significant safety concerns. Like many thermoplastic polymers, polyester exhibits melt-dripping behavior, characterized by the softening and dripping of molten material. Upon exposure to flame, polyester tends to generate melt droplets, which may either remove polymer from the burning region and stop combustion or ignite nearby materials and promote the spread of fire [3].

To address this issue, various flame retardants (FRs) have been developed for polyester fabrics, including organohalogens, metal hydroxides, and organic and inorganic compounds [2,4]. Although organohalogen flame retardants are highly effective, their application has been increasingly restricted due to well-documented environmental and health concerns. These compounds are often persistent and bioaccumulative, and their combustion or thermal degradation can generate toxic and corrosive gases, as well as highly hazardous by-products such as dioxins and furans. In addition, growing regulatory pressure and legislative restrictions, particularly in Europe and other regions, have significantly limited the use of halogenated flame retardants in textiles and consumer products [5,6]. As a result, halogen-free flame-retardant systems have attracted considerable attention. These alternatives include compounds containing nitrogen, silicon, sulfur, and boron, but the majority are phosphorus-based FRs, which are widely applied to wood, plastic, paper, and textiles. Despite their high flame-retardant efficiency, phosphorus-based systems applied to textiles often suffer from limited washing durability due to poor fixation to polymer substrates and the migration or leaching of low-molecular-weight species during laundering. Durability has long been recognized as a mandatory requirement for textile flame retardants and has been successfully achieved in conventional cotton systems through the chemical grafting of phosphorus–nitrogen flame retardants, such as Proban^®^ and Pyrovatex^®^, onto cellulose hydroxyl groups using resin-assisted fixation [7]. These considerations highlight the importance of improving the durability and fixation of phosphorus-based systems when applied to synthetic fibers such as PET [7]. Such systems can be tailored to function in the gas phase, condensed phase, or both. Compounds containing nitrogen, silicon, sulfur, and boron are known to exhibit intrinsic flame-retardant activity, and when incorporated into phosphorus-based systems, they often act synergistically to enhance overall performance [8].

Intumescent flame retardants (IFRs) have emerged as promising halogen-free systems that improve fire safety while reducing environmental impact. IFRs typically comprise a carbon source, an acid source, and a blowing agent. Upon heating, the acid source decomposes to form mineral acids that catalyze dehydration of the carbon source and promote carbonaceous char formation. Simultaneously, decomposition of the blowing agent releases gases that expand the char, producing a thick, multicellular insulating barrier between the heat source and the substrate. This barrier suppresses further burning and can also reduce melt dripping, smoke, and toxic gas release [9,10].

Nitrogen- and phosphorus-based compounds are well known for their IFR activity [9,10]. Water-based polyelectrolyte solutions containing chitosan, pectin, and mono ammonium phosphate were applied onto enzyme/corona-treated polyester using layer-by-layer (LbL) deposition, resulting in self-extinguishing performance in vertical flame testing [4]. PET fabrics were grafted with acrylamide (AM), followed by coating of polyethyleneimine (PEI) and sodium alginate (OSA), and finally cross-linked with hypophosphorous acid (HA). The treated fabric exhibited enhanced thermal stability, increased char residue, and higher decomposition temperatures. Fabrics with 15 bilayers of HA cross-linked coating self-extinguished without melt-dripping and retained durability after 12 laundering cycles [11]. In another study, maleic acid was grafted onto the PET backbone to react with pentaerythritol phosphate urea salt (PEPAS), forming a compact structure that improved flame retardancy and anti-dripping properties. The treated fabric reached a maximum LOI of 29.3, showed self-extinguishing behavior, and maintained an LOI of 26.8 after 20 laundering cycles, with minimal impact on tensile strength [12].

Silicon-containing FRs, such as polyhedral oligomeric silsesquioxane (POSS), have also been shown to promote flame retardancy [13,14]. For instance, DOPO-functionalized POSS promoted char formation in PET and decomposed to generate free radicals that acted as scavengers. DOPO-POSS improved flame retardancy by functioning simultaneously in both gaseous and condensed phases [13,14]. More recently, a combination of phytic acid and (3-piperazinylpropyl)methyldimethoxysilane produced a coating that eliminated melt-dripping, improved LOI, and reduced both the peak heat release rate (PHRR) and the total heat release (THR) of PET fabrics [15]. The PHRR represents the maximum rate of heat release during combustion and is a critical parameter for fire safety, as a lower PHRR indicates reduced fire intensity and slower fire growth. Phenyl/vinyl polysilsesquioxane (VPPSQ) was incorporated into PET/aluminum diethylphosphinate (ADP) composites, resulting in excellent flame retardancy with higher LOI values and reduced heat release, smoke, and dripping. Aluminum diethylphosphinate acted primarily in the gas phase during early combustion, whereas VPPSQ stabilized the char layer through silicon fragments, SiO_2_, and graphitized structures, thereby improving thermal stability and barrier performance [16].

Phosphorus–nitrogen–silicon flame retardants may provide superior performance compared to systems that contain only two of these elements. In such systems, phosphorus promotes char formation, nitrogen contributes by diluting oxygen and expanding the char through the release of inert gas, and silicon stabilizes the char layer, thereby enhancing thermal resistance [8]. Epoxy resins containing phosphorus–nitrogen–silicon organic/inorganic flame retardants have also demonstrated strong synergistic effects [17].

Although sericin (SC), a silk-derived protein rich in nitrogen, has been extensively investigated as a functional finishing agent for polyester textiles—particularly for enhancing hydrophilicity [18,19], UV protection [20,21], antistatic properties [21,22], and dyeability [23,24],—its application has largely been confined to comfort enhancement and surface modification. The use of sericin to improve flame-retardant performance has been reported for cotton substrates; for example, a sericin-based IFR system was chemically grafted onto cotton fabric, achieving a limiting oxygen index (LOI) of 30.57% along with good washing durability [19]. To the best of our knowledge, the use of sericin in IFR systems for PET fabrics has not yet been systematically investigated.

In the present study, an eco-friendly intumescent coating was developed for PET fabrics based on the phosphorus–nitrogen–silicon (P/N/Si) synergistic concept. The coating incorporated SC, a silk-derived protein that serves as a nitrogen source, and trisilanol isobutyl-POSS (Si-FR), a hybrid organic–inorganic particle with a silica-like cage structure and reactive silanol groups, to achieve synergy with the aluminum diethylphosphinate-based flame retardant (Exolit^®^ OP 1400, denoted as P-FR). The treated fabrics were characterized by Fourier transform infrared (FTIR) spectroscopy, scanning electron microscopy coupled with energy-dispersive X-ray spectroscopy (SEM–EDS), tensile testing, and bending length analysis. Thermal stability and flame-retardant performance were assessed using thermogravimetric analysis (TGA), limiting oxygen index (LOI), and the UL 94 vertical burning test (UL 94 VBT). Washing durability was also evaluated.

## 2. Materials and Methods

### 2.1. Materials

**Figure 1:**
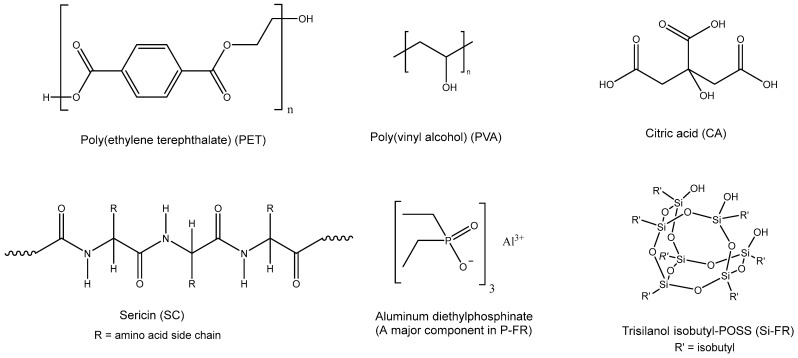
Chemical structures of the main components used in this study.

A 100% poly(ethylene terephthalate) (PET) plain-woven fabric (265 g/m^2^; warp density: 17 ends/cm; weft density: 10 picks/cm) was purchased from Thai Numchoke Textile Co., Ltd, Samut Prakan, Thailand. Sericin (crude protein content 90%, abbreviated as SC) was supplied by Kaewluang Co., Ltd., Pathum Thani, Thailand. Poly(vinyl alcohol) (Mowiol^®^, 98.0–98.8 mol% hydrolysis, MW = 195,000; abbreviated as PVA) was obtained from Sigma-Aldrich, Missouri, USA. A commercial phosphorus-based flame retardant, Exolit^®^ OP 1400, with aluminum diethylphosphinate as the major component (abbreviated as P-FR), was purchased from Clariant, Pratteln, Switzerland. Trisilanol isobutyl-POSS (SO1450, abbreviated as Si-FR) was obtained from Hybrid Plastics Inc., Hattiesburg, MS, USA. Citric acid (99.5%, abbreviated as CA) was supplied by Sigma-Aldrich. BYK-P104S, a commercial wetting and dispersing agent composed of an unsaturated polycarboxylic acid polymer and a polysiloxane copolymer, was purchased from BYK-Chemie GmbH, Wesel, Germany. Tween 60 surfactant was purchased from Acros Organics, Geel, Belgium. The chemical structures of the main components used in this study are shown in Figure 1.

### 2.2. Pretreatment of PET via Alkaline Hydrolysis

PET fabric was cut into rectangular pieces (21 cm × 29.7 cm), and the edges were overlocked to prevent fraying. The fabric samples were immersed in a 1 M NaOH solution and treated at 90 °C for 30 min under continuous magnetic stirring. To ensure uniform hydrolysis, the samples were gently turned every 10 min during treatment. After the reaction, the fabrics were thoroughly rinsed with deionized (DI) water until neutral pH was reached and then air-dried.

### 2.3. Preparation of the FR Coating Dispersions

A PVA stock solution was prepared by dissolving 30.63 g of PVA powder in 350 mL of DI water. The mixture was stirred at 80 °C on a hotplate stirrer until completely dissolved and then allowed to stand overnight at room temperature. CA (0.8 g), SC (0.4 g), dispersing agent (0.96 g), and surfactant (0.08 g) were first dissolved in 8 mL of DI water under magnetic stirring. Subsequently, 32 mL of the previously prepared PVA solution was added to obtain a volume of approximately 40 mL. The mixture was homogenized at 4000 rpm for 3 min using a high-speed homogenizer (IKA, Staufen, Germany, T25 digital ULTRA-TURRAX) to ensure uniform pre-dispersion of the components. The required amounts of P-FR and Si-FR were then gradually incorporated under continuous stirring. The resulting mixture was further sonicated in an ultrasonic bath (Elma Elmasonic Easy, Singen, Germany, 750 W) for 15 min to eliminate particle agglomeration and obtain a homogeneous coating dispersion. The coating formulations are summarized in Table 1.

### 2.4. Application of the FR Dispersion onto Polyester Fabric

The PET fabric was mounted on a stenter frame and held under tension. A 25 g portion of FR dispersion was poured along one edge of the fabric and manually spread using the knife coating technique to ensure uniform coverage across the fabric surface. The coated fabric was dried at 120 °C for 5 min on each side, followed by curing at 120 °C for 20 min.

### 2.5. Characterization

#### 2.5.1. Morphological Study

The morphology of the fabrics was examined using a Hitachi, Tokyo, Japan, Model SU5000 Schottky field-emission scanning electron microscope (FE-SEM) operated at 15.0 kV. Samples were sputter-coated with gold at 15 mA for 60 s before observation. Elemental analysis was performed using the attached energy-dispersive X-ray spectroscopy (EDS) system.

#### 2.5.2. Fourier Transform Infrared (FTIR) Spectroscopy

FTIR spectra of the fabrics were recorded using a spectrometer (Nicolet iS50, Thermo Scientific, Massachusetts, USA) over the wavenumber range of 400–4000 cm^−1^, with 64 scans obtained at a resolution of 4 cm^−1^. Measurements were performed in the attenuated total reflectance (ATR) mode with a diamond crystal.

#### 2.5.3. Thermogravimetric Analysis (TGA)

The thermal stability of the fabrics was assessed using a thermogravimetric analyzer (TGA/DSC3, Mettler Toledo, Greifensee, Switzerland) under air. Measurements were performed over a temperature range of 25 °C to 800 °C at a heating rate of 10 °C/min.

#### 2.5.4. Flammability Behavior of Treated Polyester Fabrics

The flammability of the treated polyester fabrics was evaluated using the UL 94 VBT. Specimens (5 cm × 185 mm) were mounted vertically and exposed to a flame for two consecutive applications. The evaluation criteria included afterflame time following both the first and second applications (T1 and T2), the total afterflame time for a set of five specimens (T1 + T2), afterflame plus afterglow time (T2 + T3), flame or afterglow propagation, dripping behavior, and the ignition of a cotton indicator positioned below the specimen. Based on these criteria, materials were classified into categories such as V-0, V-1, V-2, or not rated, reflecting their flame retardancy performance, as summarized in Table 2.

#### 2.5.5. Tensile Test

The tensile properties of the samples were evaluated using a universal testing machine (Tinius Olsen, Pennsylvania, USA, Model 50ST). Tests were conducted according to ASTM D5035 [25], using specimen strips (15.24 cm × 2.54 cm), a crosshead speed of 300 mm/min, and a gauge length of 75 mm. Measurements were performed in both the warp and weft directions, with five replicates for each direction. Results are presented as mean values with standard deviations.

#### 2.5.6. Bending Length Measurement

The bending length of the fabric samples was measured to evaluate their stiffness according to JIS L 1096:1999 Method A (45° Cantilever Method) [26]. Fabric specimens were allowed to overhang from a horizontal platform and gradually advanced until the leading edge reached the 45° line under its own weight. The overhang length was recorded and used to calculate the bending length and flexural rigidity. Measurements were conducted in both warp and weft directions. 

#### 2.5.7. Wash Fastness Test

The wash fastness of the flame-retardant coated samples was evaluated according to the AATCC Test Method 61-1A [27], simulating five repeated low-temperature hand-laundering cycles. The test involved washing each sample at 40 °C for 45 min with 10 steel balls in 200 mL of a 0.37% standard detergent solution.

#### 2.5.8. Limiting Oxygen Index (LOI) Test

The limiting oxygen index (LOI) of the specimens was measured according to ASTMD2863-06A [28]. Specimens (5 cm × 10 cm) were mounted vertically in a supporting frame and ignited at the top edge. LOI was defined as the lowest oxygen concentration in an oxygen/nitrogen mixture at which the specimen sustained burning for at least 3 min or until the flame propagated along the full sample length. Reported LOI values represent the average of five repeated measurements.

## 3. Results

### 3.1. Morphological Observation of the PET Surface Before and After Alkaline Hydrolysis

**Figure 2:**
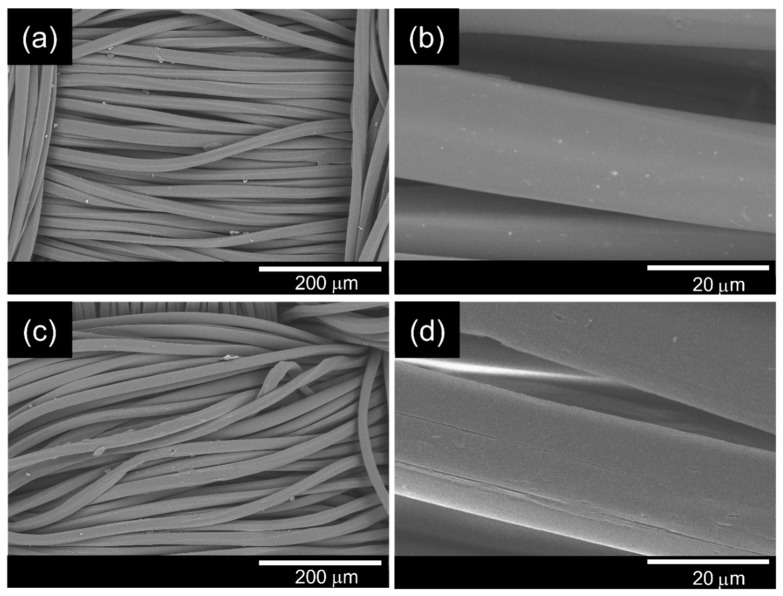
SEM images of the PET fabric before (**a**,**b**) and after alkaline treatment with 1.0 M sodium hydroxide (**c**,**d**).

SEM images (Figure 2) showed that the untreated PET fibers had a smooth and compact surface. After treatment with 1.0 M NaOH at 90 °C for 30 min, slight surface roughening and localized etching were observed. These morphological changes are consistent with alkaline hydrolysis of PET, which involves preferential cleavage of ester bonds in the amorphous regions, leading to surface erosion. The hydrolysis reaction introduces additional hydroxyl and carboxyl end groups on the fiber surface, thereby increasing surface polarity and hydrophilicity, which facilitates subsequent coating adhesion.

The surface modification was further confirmed by water contact angle measurements. Following alkaline hydrolysis, the contact angle decreased from 129.44 ± 3.69° (untreated PET) to 118.18 ± 3.35°. Statistical analysis (one-way ANOVA followed by Tukey’s HSD test) confirmed that this reduction was significant (*p* < 0.05), indicating enhanced surface wettability after treatment.

### 3.2. Morphological Observation of the PET Surface Coated with P-FR and SC

**Figure 3:**
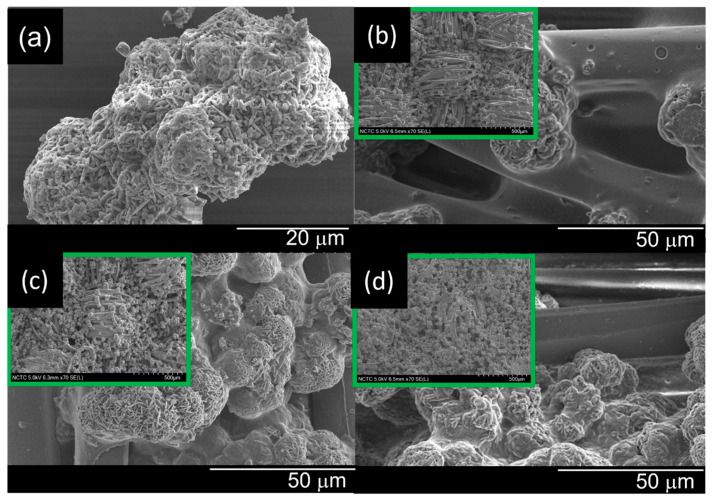
SEM images of (**a**) P-FR particles and (**b**) OP5-SC, (**c**) OP10-SC, and (**d**) OP15-SC fabrics.

P-FR has been widely used to improve the fire resistance of polyamides and PET [16], functioning through simultaneous action in both gas and condensed phases [29]. From Figure 3a, P-FR particles appeared as irregularly shaped agglomerates with rough, uneven surfaces, mostly smaller than 53 μm [30].

Figure 3b–d present SEM images of fabrics coated with 1% *w*/*v* SC and P-FR at concentrations of 5, 10, and 15% *w*/*v* in the coating formulation. The P-FR particles were observed on the fiber surfaces, with localized aggregation at higher loadings. As the P-FR concentration increased, a higher particle density was observed on the fiber surfaces.

### 3.3. FTIR Spectra of Fabrics Treated with P-FR and SC

**Figure 4:**
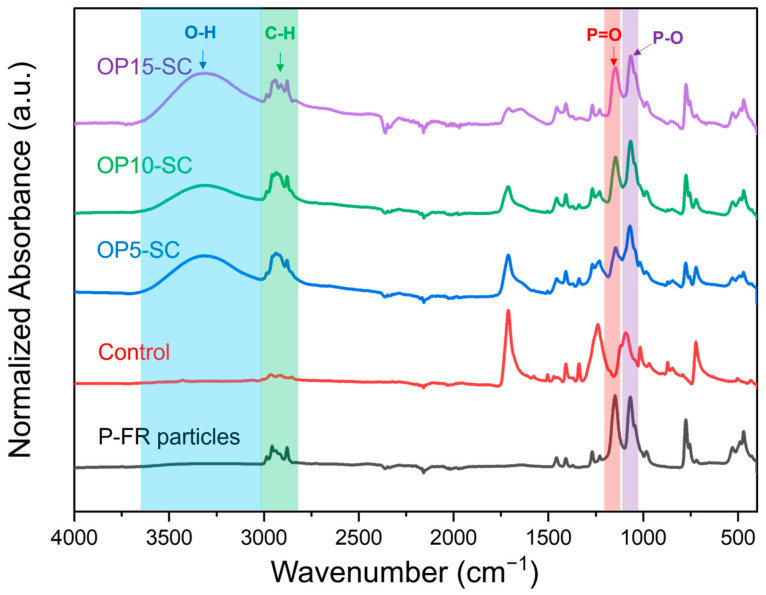
FTIR spectra of P-FR particles, control fabric, and fabrics treated with P-FR and SC.

According to the FTIR spectra shown in Figure 4, the spectrum of P-FR particles revealed a broad peak at 2864–2989 cm^−1^, corresponding to C-H stretching of alkyl groups. Characteristic peaks of P=O and P-O appeared at 1179 and 1021 cm^−1^, respectively [31]. These peaks were also observed in P-FR-treated fabrics (OP5-SC, OP10-SC, and OP15-SC). The peak at 1721 cm^−1^ in the control fabric corresponded to C=O stretching of the aromatic ester. This peak appeared less pronounced in the treated fabrics because the P-FR particles partially covered the fabric surfaces. Broad O-H stretching peaks in the range of 3200–3600 cm^−1^ corresponded to the hydroxyl groups of PVA and CA, which were used as binder and crosslinker, respectively. These observations confirm the successful coating of P-FR on the PET fabrics.

### 3.4. Effects of P-FR and SC on the Flammability of PET Fabrics

Table 3 presents the results of the UL 94 VBT. As expected, the uncoated fabric did not achieve any classification, as it continued to burn beyond the allowable time limits and produced molten drips. Similarly, PET fabric treated with 5 wt% P-FR (OP5) failed to pass the UL 94 VBT due to excessive afterflame times, which were attributed to the insufficient phosphorus content that did not provide effective flame retardancy. The OP10 sample achieved a V-1 rating, whereas the OP15 sample attained a V-0 rating. OP10 exhibited slightly longer afterflame times than OP15. In contrast, OP15, with its higher phosphorus content, demonstrated superior flame-retardant performance, characterized by shorter afterflame times and the absence of melt dripping, consistent with the V-0 classification.

To evaluate the potential phosphorus/nitrogen (P/N) synergistic effects, the influence of incorporating 1% *w*/*v* SC into a flame-retardant coating containing 10% *w*/*v* P-FR as the primary component was investigated. The evaluation included a flammability test using the UL 94 VBT and morphological characterization of the combustion residue by SEM. Fabrics treated with SC alone (1% *w*/*v*; OP0-SC) or P-FR alone (10% *w*/*v*; OP10) were compared with those treated with both SC and P-FR (OP10-SC), as summarized in Table 4.

As shown in Table 4, the SC-treated fabric (OP0-SC) failed all UL 94 test criteria, exhibiting a total afterflame time of 278.9 s, which was even higher than that of the uncoated sample (213.3 s). This result indicates that although SC contains nitrogen, it is a highly combustible organic protein with limited char-forming ability. In contrast, the incorporation of P-FR with SC (OP10-SC) substantially reduced the afterflame time to 23.0 s and produced self-extinguishing behavior without melt dripping, resulting in a V-0 rating. OP10 (without SC) achieved only a V-1 rating, with a longer afterflame time of 35.6 s and visible melt dripping. These findings suggest that SC enhanced flame retardancy only in the presence of P-FR. The reduction in afterflame time indicates higher resistance to flame propagation compared to samples lacking SC.

### 3.5. Morphological Observation of Char Residues Obtained from Samples Coated with P-FR and SC

**Figure 5:**
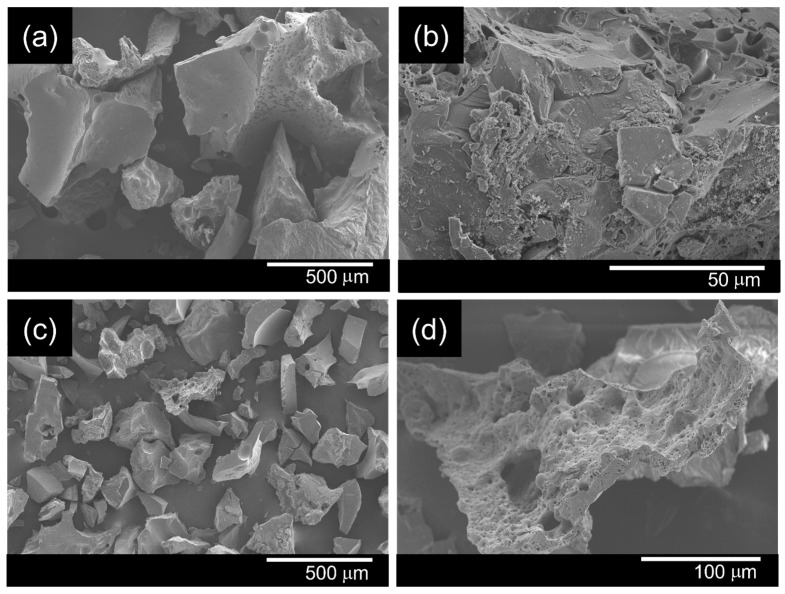
SEM images of char residues after UL 94 VBT for OP10 (**a**,**b**) and OP10-SC (**c**,**d**).

As shown in the SEM images in Figure 5, the char of the OP10 sample exhibited fewer pores than that of OP10-SC, confirming that SC interacted with phosphorus to produce a more porous char structure. The expanded char layer acted as an effective barrier against heat and oxygen [32], reducing flame propagation more efficiently than P-FR alone.

### 3.6. Morphological Observation of the PET Surface Coated with P-FR, SC, and Si-FR

**Figure 6:**
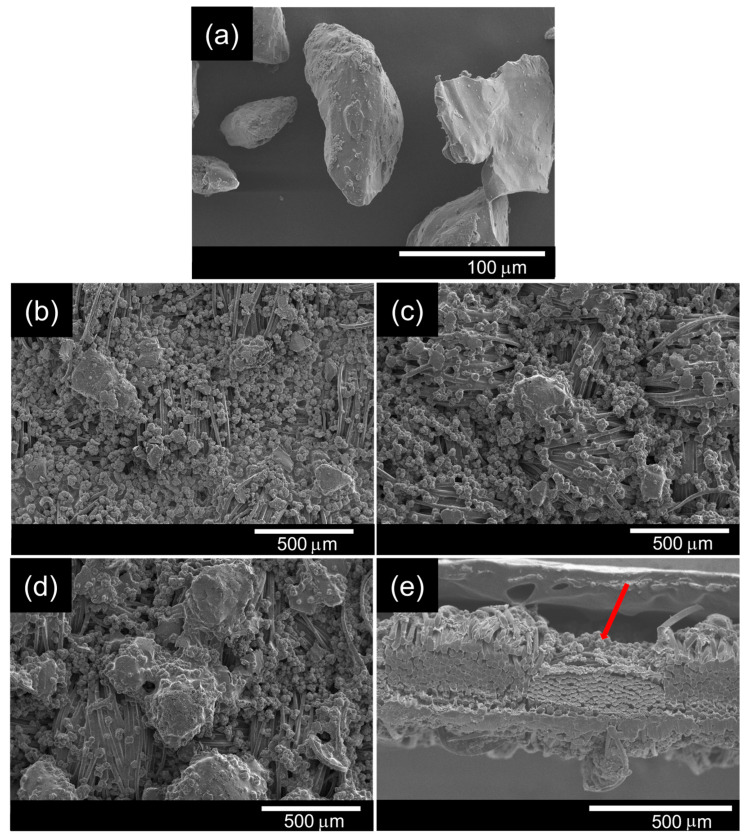
SEM images showing the top view of (**a**) Si-FR particles, (**b**) OP10-SC-Si1, (**c**) OP10-SC-Si2, and (**d**) OP10-SC-Si3, and (**e**) the cross-sectional view of OP10-SC-Si2, showing coating deposits on and between fibers (arrow).

The influence of Si-FR on flame-retardant performance was examined in samples coated with P-FR (10% *w*/*v*) and SC (1% *w*/*v*) to investigate its potential contribution to P/N/Si synergism. SEM images of Si-FR particles and the surface of PET fabrics coated with different Si-FR concentrations are presented in Figure 6.

The morphological differences between P-FR and Si-FR flame retardants were clearly observed in the SEM images. Si-FR particles (Figure 6a) exhibited relatively smooth surfaces with irregular shapes and reached sizes up to 100 µm, whereas P-FR particles (Figure 3a) appeared as irregularly shaped agglomerates forming clusters with sizes of several tens of micrometers. Figure 6b–d reveal that both P-FR and Si-FR particles were densely distributed across the fabric surface, and the amount of Si-FR adhered to the fabric increased with the applied Si-FR content. To further clarify the coating distribution, a cross-sectional SEM image of OP10-SC-Si2 is shown in Figure 6e. The image indicates the presence of coating materials deposited on the fiber surfaces.

**Figure 7:**
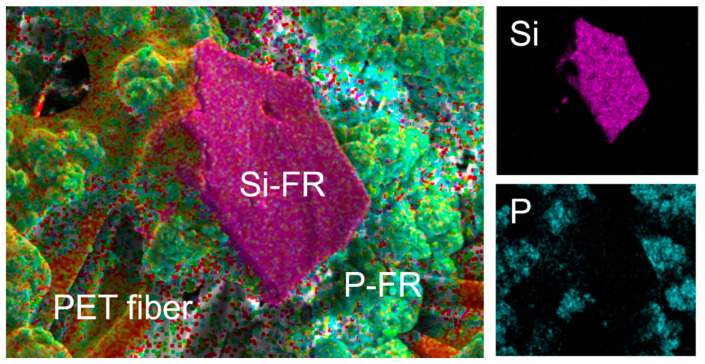
EDS elemental mapping images of OP10-SC-Si2 showing the distribution of P and Si.

Figure 7 presents the EDS elemental mapping images of OP10-SC-Si2. As expected, silicon was detected in regions corresponding to Si-FR particles, while phosphorus was identified in smaller particles corresponding to P-FR.

### 3.7. FTIR Spectra of the Fabrics Treated with P-FR, SC, and Si-FR

**Figure 8:**
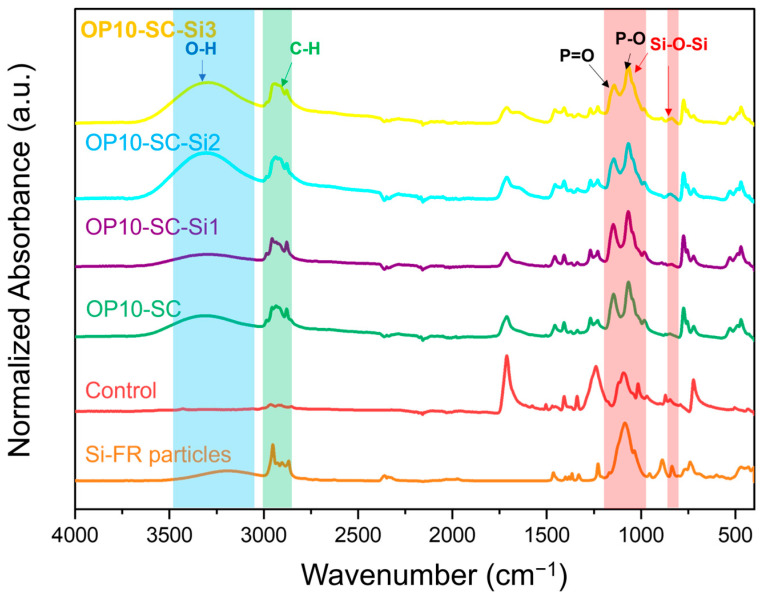
FTIR spectra of Si-FR particles, control fabric, and fabrics treated with P-FR, SC, and Si-FR.

FTIR analysis was conducted to confirm the presence of Si-FR on the fabric samples, as shown in Figure 8. Si-FR exhibited a broad band at 3020–3500 cm^−1^, corresponding to O–H stretching vibrations of silanol groups. Since the Si-FR structure contains only three hydroxyl groups, this peak was not particularly prominent. Peaks observed at 2859–2980 cm^−1^ were attributed to C–H stretching vibrations of the isobutyl groups. A strong and distinct peak in the range of 983–1178 cm^−1^ corresponded to Si–O–Si asymmetric stretching vibration [33], while peaks at 809–847 cm^−1^ were assigned to Si–O–Si symmetric stretching vibrations [34]. In the FTIR spectra of fabric samples treated with Si-FR and P-FR, the Si–O–Si asymmetric stretching peak was masked by the P=O peak at 1179 cm^−1^ due to the higher P-FR loading. The Si–O–Si symmetric absorption peak was weak but still detectable in the sample with high Si-FR loading (OP10-SC-Si3).

### 3.8. Effects of Coatings Comprising P-FR, SC, and Si-FR on the Flammability of PET Fabrics

**Figure 9:**
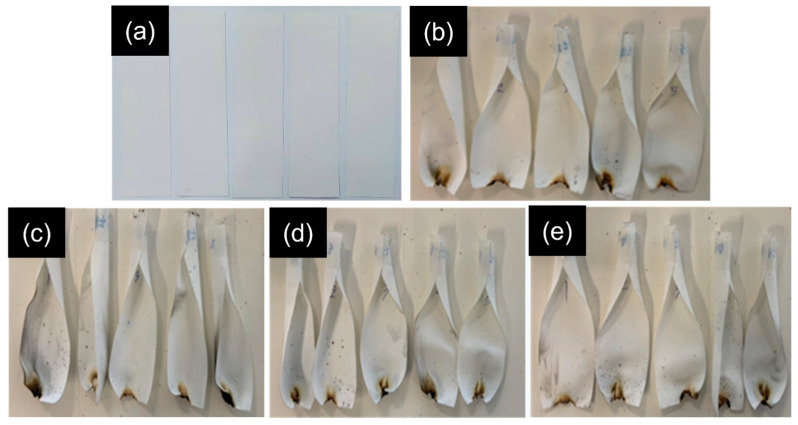
Photographs of (**a**) alkaline-treated PET fabrics before burning and (**b**–**e**) fabrics after UL 94 VBT: (**b**) OP10-SC; (**c**) OP10-SC-Si1; (**d**) OP10-SC-Si2; and (**e**) OP10-SC-Si3.

From Table 5, the UL 94 VBT results of the PET fabrics treated with P-FR, SC, and Si-FR indicated that all samples achieved a V-0 rating. Previous studies have reported that POSS, when incorporated into a phosphorus-based intumescent system, can produce a more compact and mechanically robust char [16]. In the present study, the addition of 2–3% Si-FR to the FR coating slightly reduced the afterflame time. In contrast, the incorporation of a low Si-FR concentration (1% *w*/*v*) was insufficient to reinforce the char. The sample OP10-SC-Si2 was selected for further investigation using TGA and LOI. Photographic images of the samples after UL 94 VBT are shown in Figure 9.

### 3.9. Effects of Coatings Comprising P-FR, SC, and Si-FR on the Thermal Stability of PET Fabrics

**Figure 10:**
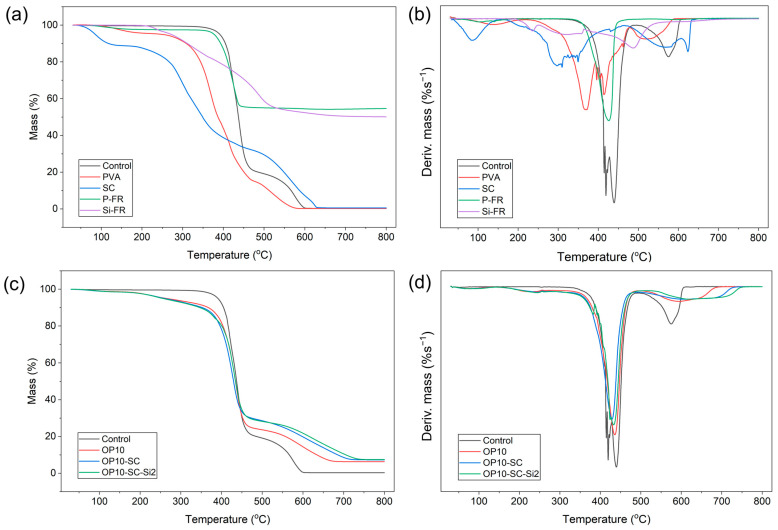
TGA and DTG thermograms of (**a**,**b**) coating components and (**c**,**d**) treated fabrics.

Figure 10a,b presents the TGA and DTG thermograms of additives incorporated into flame-retardant coatings on PET fabrics. All additives exhibited an initial minor weight loss below approximately 200 °C, primarily due to the evaporation of the adsorbed moisture.

SC and PVA displayed relatively low thermal stability compared to P-FR and Si-FR. SC showed the earliest onset of degradation, with T_−10%_ (temperature corresponding to 10% mass loss) recorded at 118.2 °C, associated with moisture loss. A pronounced mass loss occurred during the first decomposition stage, with a maximum temperature (T_max_) of 296.5 °C and a corresponding weight loss of 30.3%. The residual mass was negligible (0.6%) at 800 °C, indicating nearly complete volatilization of organic matter. PVA, used as a binder, exhibited improved stability compared with SC, with an initial moisture loss at 142.5 °C and T_−10%_ at 309.0 °C. Nevertheless, its residual mass remained minimal (0.2%), suggesting that coating with SC and PVA did not provide condensed-phase protection.

In contrast, the flame-retardant additives P-FR and Si-FR exhibited significantly higher thermal stability and greater residual masses. P-FR showed the highest thermal stability, with T_−10%_ at 393.3 °C and a single major decomposition peak at 426.0 °C. Its residue mass at 800 °C was 54.7 wt%, attributed to the formation of inorganic phosphate and aluminum–phosphate networks. Si-FR exhibited multi-step degradation with a T_−10%_ value of 311.7 °C. Although its initial decomposition occurred at a lower temperature than P-FR, the degradation proceeded through multiple, less abrupt stages, yielding a final residue mass of 50.1 wt%, primarily associated with silica-based structures.

Figure 10c,d compares the TGA and DTG thermograms of the fabric samples, with the corresponding data summarized in Table 6. The TGA and DTG thermograms of fabric samples are compared, with the corresponding data summarized in Table 6. The control fabric displayed two major decomposition stages, with T_max_ at 439.5 °C and 575.2 °C and T_−10%_ at 404.8 °C. The residual mass at 800 °C was only 0.3 wt%, confirming that PET predominantly decomposed into volatile products such as CO, CO_2_, and low-molecular-weight hydrocarbons under oxidative conditions. Incorporating flame retardants significantly altered the degradation profile of PET fabrics. Samples treated with P-FR (OP10, OP10-SC, and OP10-SC-Si2) exhibited three distinct degradation stages, with an additional stage at approximately 242–245 °C corresponding to the early decomposition of additives (PVA, SC, Si-FR). Furthermore, the onset degradation temperature decreased to 347–368 °C due to the catalytic effect of P-FR, which promoted earlier PET chain scission and facilitated char formation. The reduced T_−10%_ therefore reflects a shift in the degradation pathway toward condensed-phase flame-retardant action rather than diminished thermal stability. Correspondingly, flame-retardant-treated samples exhibited substantially higher residues, increasing from 0.3 wt% in untreated PET to 6.3–7.5 wt% in treated fabrics, confirming enhanced condensed-phase protection.

It should be emphasized that SC does not increase the intrinsic thermal stability of the material. The low T_−10%_ value of SC primarily reflects moisture loss and the inherent thermal behavior of the protein structure. Meanwhile, the decreased T_−10%_ observed in the coated fabrics arises from the catalytic effect of P-FR, which accelerates dehydration and promotes condensed-phase charring. Thus, SC contributes to the stabilization of the resulting char structure through synergistic interaction with phosphorus species, rather than by improving the initial thermal stability.

Above 500 °C, untreated PET exhibited a sharp oxidation peak associated with rapid char combustion, whereas flame-retardant-treated fabrics showed delayed and suppressed oxidation peaks. The third-stage peak shifted toward higher temperatures, reaching 623.3 °C for OP10-SC-Si2, compared with 611.5 °C for OP10-SC, 596.2 °C for OP10, and 575.2 °C for the control. This shift, along with reduced peak intensity, indicated that SC and Si-FR contributed to the formation of thermally stable char layers that suppressed oxidative degradation. The mass loss rate at T_max_ also decreased in treated samples during the second degradation stage; untreated PET exhibited a rate of −1.71%/°C, whereas the treated samples ranged from −1.41 to −1.26%/°C. Similarly, the third-stage char oxidation rate decreased from −0.36%/°C in untreated PET to −0.14 to −0.12%/°C in treated fabrics, confirming the improved thermal stability of the char.

These thermal degradation behaviors support an intumescent flame-retardant mechanism facilitated by the synergistic interaction of phosphorus (from P-FR), nitrogen (from SC), carbon sources (from PVA and PET), and silicon (from Si-FR). Phosphorus-based species generated polyphosphoric acid, catalyzing the dehydration of PVA and PET to form carbonaceous char. Concurrently, SC released inert nitrogen-containing gases that expanded the matrix and created a porous, insulating barrier. Si-FR further reinforced the structural integrity of this protective layer through the formation of silica or silicate phases, as evidenced by the highest final residue (7.5 wt%) and the delayed char oxidation peak observed in OP10-SC-Si2. Together, these effects produced a stable, thermally resistant intumescent layer that suppressed heat transfer and volatile release.

### 3.10. Effects of Coatings Comprising P-FR, SC, and Si-FR on the Mechanical Properties of PET Fabrics

As seen in Table 7, the tensile test results indicate that the incorporation of P-FR and Si-FR exerted a reinforcing effect on PET fabrics. In the warp direction, OP10-SC and OP10-SC-Si2 exhibited increases in tensile strength of approximately 15.2% and 22.6%, respectively, compared with the untreated fabric. Statistical analysis confirmed that the tensile strength of OP10-SC-Si2 was significantly higher than that of OP10-SC (*p* < 0.05), demonstrating that the incorporation of Si-FR contributed to further reinforcement compared with the P-FR/SC system. This enhancement can be attributed to the formation of a more compact and crosslinked Si–O–Si network, which improves stress transfer and structural integrity along the aligned warp yarns.

In contrast, tensile strength in the weft direction was not significantly affected by the coating formulation. This behavior may be related to the structural difference between yarn systems, as warp yarns are typically more stretched and aligned, whereas weft yarns are more crimped. Consequently, the coating can penetrate and adhere more effectively to warp yarns than to weft yarns.

The application of coatings (OP10-SC and OP10-SC-Si2) reduced yarn mobility by bridging adjacent yarns, leading to decreased elongation in both directions. In the warp direction, elongation decreased by nearly 50% relative to the untreated fabric, while in the weft direction, elongation was reduced by approximately 36%. Notably, although Si-FR further enhanced tensile strength, it did not significantly alter elongation compared to OP10-SC.

### 3.11. Effects of Coatings Comprising P-FR, SC, and Si-FR on the Bending Length of PET Fabrics

As seen in Table 8, the bending length results indicate that the coating treatment significantly affected the mechanical behavior of PET fabrics. One-way ANOVA revealed a statistically significant effect of coating formulation on bending length in both warp and weft directions (*p* < 0.05). Tukey’s post hoc test confirmed that all coated samples exhibited significantly higher bending length than the untreated control.

In the warp direction, OP10-SC showed significantly higher bending length than OP10, whereas no significant difference was observed between OP10 and OP10-SC-Si2. In the weft direction, OP10-SC-Si2 exhibited significantly higher values than OP10 and OP10-SC. The untreated PET displayed the lowest bending length, reflecting its inherent flexibility. The increase after coating is attributed to restricted yarn mobility resulting from the formation of a surface coating layer. The addition of SC further enhanced bending length, particularly in the warp direction, likely due to strengthened intermolecular interactions and densification of the coating matrix.

Although a slight increase in add-on was observed from OP10 to OP10-SC and OP10-SC-Si2, these differences were not statistically significant (*p* > 0.05), indicating that bending behavior was not governed solely by coating quantity. Instead, coating chemistry and network structure appear to play a more decisive role.

The measured bending length values (≈41–63 mm) fall within a moderate stiffness range, suggesting that the treated fabrics remain suitable for interior and technical textile applications requiring dimensional stability while maintaining acceptable fabric handle.

### 3.12. Wash Durability of OP10-SC-Si2

The wash durability of OP10-SC-Si2 was evaluated, and the results are summarized in Table 9. Before laundering, the coated fabric achieved a V-0 classification in the UL 94 VBT, with a total afterflame time (T_1_ + T_2_) of 23.1 s. After five washing cycles, the classification decreased to V-2 and the T_1_ + T_2_ value increased to 41 s. The coating weight loss after five cycles was 34.5 ± 0.4 wt%, calculated relative to the initial add-on, corresponding to an overall mass reduction of approximately 7.14 ± 0.3 wt% based on the total fabric weight. After 50 laundering cycles, T_1_ + T_2_ further increased to 131.4 s, indicating progressive deterioration of flame-retardant performance upon repeated washing. This decline can be attributed to mechanical agitation and chemical exposure during laundering, which likely caused partial removal of loosely bound flame-retardant components. The decrease in coating retention weakened the integrity of the condensed-phase protective layer and reduced char cohesion, thereby prolonging afterflame time and promoting melt dripping.

Nevertheless, even after laundering, the coated fabrics maintained self-extinguishing behavior but produced flaming drips, resulting in a V-2 classification. This indicates that residual gas-phase flame inhibition and condensed-phase protection were sufficient to suppress flame propagation. However, the anti-dripping performance—closely related to char continuity and melt viscosity—was significantly compromised after repeated washing.

### 3.13. Effects of Coatings Comprising P-FR, SC, and Si-FR on the LOI Values of PET Fabrics

The limiting oxygen index (LOI) represents the minimum oxygen concentration required to sustain flaming combustion. Materials with LOI values below 21% are readily combustible, whereas values ≥ 28% are generally considered self-extinguishing under practical fire conditions [35].

Untreated PET exhibited an LOI of 21.1 ± 0.4%, indicating borderline flammability and consistent with its failure in the UL 94 VBT test. Application of 10% *w*/*v* P-FR (OP10) significantly increased the LOI to 32.2 ± 1.1%. Statistical analysis confirmed that all treated samples exhibited significantly higher LOI values than untreated PET (*p* < 0.05). However, no significant differences were observed among OP10 (32.2 ± 1.1%), OP10-SC (31.3 ± 0.2%), and OP10-SC-Si2 (31.7 ± 0.2%) before washing (*p* > 0.05), indicating that phosphorus was the dominant factor governing the critical oxygen concentration under pristine conditions. The discrepancy between UL 94 and LOI results suggests that SC primarily enhanced anti-dripping behavior and char cohesion rather than substantially altering the oxygen threshold for sustained combustion.

After five washing cycles, LOI values decreased to 28.6 ± 0.5% (OP10), 27.0 ± 0.9% (OP10-SC), and 30.5 ± 0.3% (OP10-SC-Si2) (Table 10). Significant differences emerged among the formulations after laundering (*p* < 0.01), with OP10-SC-Si2 retaining the highest LOI. Although washing reduced LOI values, all treated fabrics remained well above that of untreated PET, indicating preservation of condensed-phase flame-retardant functionality. The decline in UL 94 classification from V-0 to V-2 was therefore mainly associated with melt dripping and reduced char cohesion rather than a complete loss of flame-inhibiting capability.

Notably, despite measurable coating loss during laundering, OP10-SC-Si2 maintained superior LOI retention. This indicates that flame-retardant performance after washing depends not only on coating quantity but also on the integrity and chemical stability of the residual protective layer. The formation of a crosslinked Si–O–Si network likely enhances the stability of the remaining P/N/Si structure, thereby improving resistance to washing-induced degradation and sustaining effective flame retardancy.

### 3.14. Proposed Interaction Mechanism

**Figure 11:**
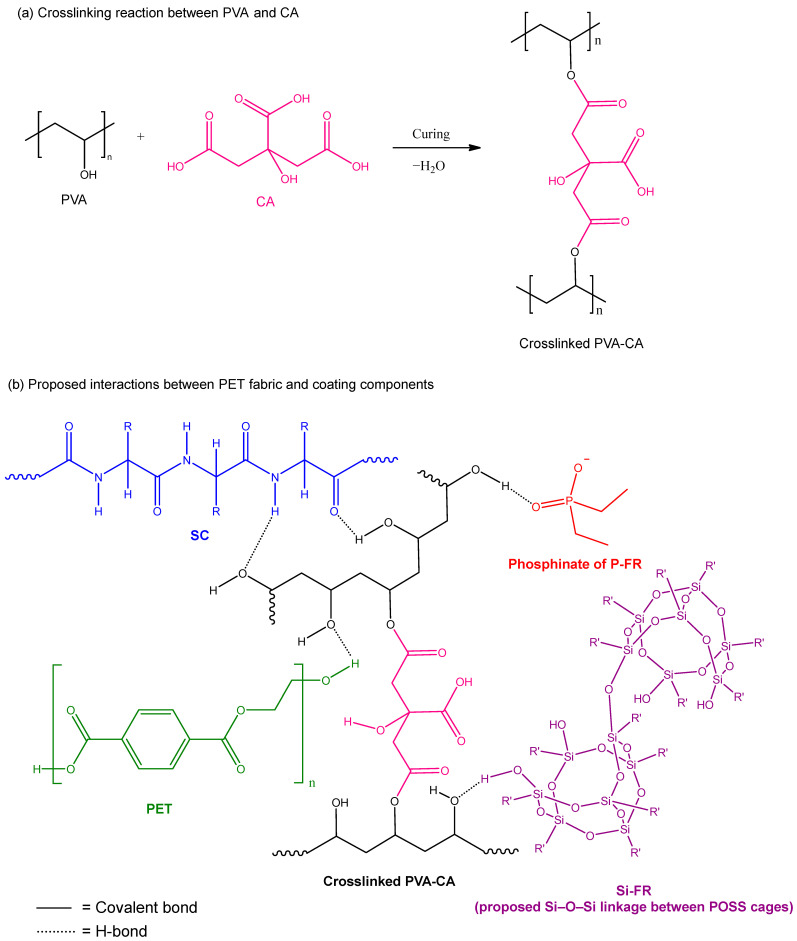
(**a**) Crosslinking reaction between PVA and CA during curing. (**b**) Proposed interactions among PET and coating components. Solid lines represent covalent bonds; dotted lines indicate hydrogen bonding.

Based on the flame-retardant performance and washing durability results, a plausible interaction mechanism for the multi-component coating system is proposed, as illustrated in Figure 11. During curing, CA reacts with PVA through esterification, forming covalent ester linkages and generating a crosslinked PVA–CA network that enhances coating cohesion and structural stability.

In addition to covalent crosslinking, hydrogen bonding is considered the primary intermolecular interaction governing the integrity of the coating system. Hydroxyl-containing components—including alkaline-treated PET, PVA, CA, SC, and Si-FR (via silanol groups)—can participate in extensive O–H···O hydrogen bonding. These interactions contribute to both interfacial adhesion between the coating and PET and internal cohesion within the coating matrix. Furthermore, the amide N–H groups of SC serve as hydrogen bond donors, while multiple carbonyl-containing groups act as hydrogen bond acceptors. Carbonyl (C=O) functionalities are present in alkaline-treated PET (ester and carboxylic groups), CA (both residual carboxylic acids and newly formed ester groups), SC (amide carbonyls), and the phosphinate structure of P-FR (P=O groups). The complementary donor–acceptor interactions among these functional groups promote the formation of a cohesive and interconnected hydrogen bonding network. Collectively, these synergistic interactions enhance coating cohesion and structural integrity, leading to improved washing durability while maintaining self-extinguishing behavior and significantly higher LOI values compared with the untreated control fabric.

### 3.15. Comparative Analysis with Phosphinate-Based Flame-Retardant Systems

To clarify the novelty and advantages of the present flame-retardant system, a comparative analysis with representative phosphinate-based flame-retardant systems reported in the literature was conducted. As summarized in Table 11, the comparison was deliberately focused on phosphinate-type systems, since aluminum diethylphosphinate (ADP), used in the present study, belongs to this family and shares similar flame-retardant mechanisms with other metal phosphinates. Phosphinate-based flame retardants are widely applied in polyester materials due to their high thermal stability, compatibility with melt-processing temperatures of PET, and their combined gas-phase and condensed-phase flame-retardant actions. It should be noted that some reported systems incorporated phosphinate flame retardants via melt blending or fiber spinning, which differ fundamentally from post-coating approaches. These systems are included for broader contextual comparison rather than direct performance equivalence.

Compared with melt-blended phosphinate systems, which typically require homogeneous dispersion within the bulk polymer matrix, the present coating strategy enables the formation of a concentrated P/N/Si-rich protective layer at the fabric surface. This structural localization enhances condensed-phase barrier efficiency without fundamentally altering the bulk mechanical integrity of PET fibers. While the coating add-on (27.34 wt%) appears higher than the phosphinate loadings reported for melt-blended systems, the active flame-retardant species are primarily confined to the fabric surface, fundamentally differing from volumetric incorporation within bulk polymers.

Bulk systems have achieved high LOI values (e.g., 32.8% in injection-molded PET); however, their specimen geometry and combustion behavior differ substantially from those of textile fabrics. Notably, the present coating system attained V-0 classification and effectively suppressed melt dripping in a flexible fabric substrate, which is inherently more challenging due to its higher surface area and open fibrous structure. Furthermore, LOI values remained above 30% after washing, despite partial coating loss, indicating retention of functional P/N/Si-rich structures. Durability data of this type were not consistently reported in several previously published phosphinate-based systems.

## 4. Conclusions

An eco-friendly intumescent flame-retardant coating for poly(ethylene terephthalate) (PET) fabrics was developed using a multi-component system comprising aluminum diethylphosphinate (P-FR), sericin (SC), and trisilanol isobutyl-POSS (Si-FR). Poly(vinyl alcohol) was used as a binder, and citric acid was used as a chemical crosslinker. PET fabrics treated with SC alone or with low concentrations of P-FR did not exhibit flame-retardant properties, underscoring the critical role of P-FR in achieving effective fire resistance. Coatings with 10% and 15% *w*/*v* P-FR yielded fabrics with V-1 and V-0 ratings, respectively, in the UL 94 vertical burning test (UL 94 VBT). Notably, the incorporation of only 1% *w*/*v* sericin into the coating with 10% *w*/*v* P-FR was sufficient to upgrade the UL 94 classification from V-1 to V-0, highlighting the strong synergistic effect of sericin even at a low loading. According to the TGA results, a coating comprising 10% P-FR, 1% SC, and 2% Si-FR lowered the onset degradation temperature (10% mass loss) from 404.8 °C to 347.0 °C while increasing the residue at 800 °C from 0.3% to 7.5%. The limiting oxygen index (LOI) values increased from 21.1 ± 0.4% for untreated PET to 31.7 ± 0.2% for treated fabrics. Tensile strength in the warp direction increased by 22.6%, whereas elongation at break was significantly reduced. These results indicate that P-FR functioned as a flame retardant through both condensed and gas phase mechanisms, whereas SC and Si-FR suppressed flame propagation, improved char integrity, and enhanced the resistance of the char to oxidation. Importantly, after 50 washing cycles, the treated fabrics maintained self-extinguishing behavior, indicating preserved flame-retardant functionality despite a decrease in UL 94 classification from V-0 to V-2 due to melt dripping. Overall, this multi-component coating improved the fire safety of PET fabrics using environmentally benign and non-toxic components. Future work will focus on mitigating melt-dripping after repeated laundering by optimizing coating cohesion and crosslink density to further enhance washing stability. In addition, cone calorimetry will be performed to quantify heat release parameters and fire growth characteristics. These investigations will provide deeper insight into long-term durability and combustion mechanisms, thereby supporting the continued development of sustainable, high-performance flame-retardant coatings for practical textile applications.

## Figures and Tables

**Table 1:** Notation of fabric samples (the control refers to PET fabric pre-treated by alkaline hydrolysis without any flame-retardant coating).

Sample Code	Coating Composition (g)			Coating Composition (% *w*/*v*)		
	SC	P-FR	Si-FR	SC	P-FR	Si-FR
Control	-	-	-	-	-	-
OP0-SC	0.4	-	-	1	-	-
OP5	-	2	-	-	5	-
OP10	-	4	-	-	10	-
OP15	-	6	-	-	15	-
OP10-SC	0.4	4	-	1	10	-
OP15-SC	0.4	6	-	1	15	-
OP10-SC-Si1	0.4	4	0.4	1	10	1
OP10-SC-Si2	0.4	4	0.8	1	10	2
OP10-SC-Si3	0.4	4	1.2	1	10	3

**Table 2:** Evaluation criteria for V-0, V-1, and V-2 classifications in the UL 94 VBT.

Criteria Condition(s)	Flammability Rating		
	V-0	V-1	V-2
Afterflame time for each individual specimen (T1 or T2)	≤10 s	≤30 s	≤30 s
Total afterflame time of a set of five specimens (T1 + T2)	≤50 s	≤250 s	≤250 s
Afterflame plus afterglow time for each individual specimen after the second flame application (T2 + T3)	≤30 s	≤60 s	≤60 s
Afterflame or afterglow reached the clamping device (125 mm)	No	No	No
Cotton indicator ignited by flaming particles or drops	No	No	Yes

**Table 3:** UL 94 VBT results for PET fabrics finished with P-FR.

UL 94 VBT Criteria	Sample			
	Control	OP5	OP10	OP15
T1 + T2 (s)	213.3	202.2	35.6	27.7
T2 + T3 ≤ 30 s	No	No	Yes	Yes
T1, T2 ≤ 10 s	No	No	No	Yes
T1, T2 ≤ 30 s	No	No	Yes	Yes
Afterflame/afterglow ≤ 125 mm	No	No	Yes	Yes
Dripping	Observed	Observed	None	None
Ignition of cotton	Yes	Yes	No	No
Classification	Not rated	Not rated	V-1	V-0

**Table 4:** UL 94 VBT results for PET fabrics treated with SC, P-FR, and their combination.

UL 94 VBT Criteria	Sample			
	Control	OP0-SC	OP10	OP10-SC
T1 + T2 (s)	213.3	278.9	35.6	23.0
T2 + T3 ≤ 30 s	No	No	Yes	Yes
T1, T2 ≤ 10 s	No	No	No	Yes
T1, T2 ≤ 30 s	No	No	Yes	Yes
Afterflame/afterglow ≤ 125 mm	No	No	Yes	Yes
Dripping	Observed	Observed	None	None
Ignition of cotton	Yes	Yes	No	No
Classification	Not rated	Not rated	V-1	V-0

**Table 5:** UL 94 VBT results of PET fabrics treated with different Si-FR concentrations (all samples also contained 10% *w*/*v* P-FR and 1% *w*/*v* SC).

UL 94 VBT Criteria	Silicon Concentration (% *w*/*v*)			
	0	1	2	3
T1 + T2 (s)	23.0	23.1	20.1	17.5
T2 + T3 ≤ 30 s	Yes	Yes	Yes	Yes
T1, T2 ≤ 10 s	Yes	Yes	Yes	Yes
T1, T2 ≤ 30 s	Yes	Yes	Yes	Yes
Afterflame/afterglow ≤ 125 mm	Yes	Yes	Yes	Yes
Dripping	None	None	None	None
Ignition of cotton	No	No	No	No
Classification	V-0	V-0	V-0	V-0

**Table 6:** TGA and DTG results of the fabric samples.

Sample	T_−10%_ (°C)	Stage 1		Stage 2		Stage 3		Residue (wt%)
		T_max_ (°C)	Rate at T_max_ (wt%/°C)	T_max_ (°C)	Rate at T_max_ (wt%/°C)	T_max_ (°C)	Rate at T_max_ (wt%/°C)	
Control	404.8	-	-	439.5	−1.71	575.2	−0.36	0.3
OP10	367.7	245.2	−0.05	435.5	−1.41	596.2	−0.14	6.3
OP10-SC	352.8	242.3	−0.05	427.8	−1.26	611.5	−0.12	7.3
OP10-SC-Si2	347.0	244.3	−0.06	432.5	−1.31	623.3	−0.12	7.5

**Table 7:** Tensile test results of the fabric samples.

Sample	Warp Direction		Weft Direction	
	Tensile Strength (N)	Elongation at Break (%)	Tensile Strength (N)	Elongation at Break (%)
Control	1511.8 ± 72.7 ^c^	61.4 ± 6.5 ^a^	1800.0 ± 94.7 ^a^	103.0 ± 21.1 ^a^
OP10-SC	1740.8 ± 57.7 ^b^	30.2 ± 1.8 ^b^	1888.7 ± 132.4 ^a^	66.2 ± 6.7 ^b^
OP10-SC-Si2	1852.8 ± 33.2 ^a^	32.6 ± 0.4 ^b^	1893.2 ± 70.5 ^a^	65.8 ± 3.8 ^b^

Data are expressed as mean ± SD (*n* = 5). Different superscript letters within the same column indicate statistically significant differences (one-way ANOVA followed by Tukey’s HSD test (*p* < 0.05).

**Table 8:** Bending length of PET fabrics measured according to JIS L 1096:1999 (Method A, 45° cantilever method) [26] and add-on (wt%) after flame-retardant coating.

Sample	Bending Length (mm)		% Add-On (wt%)
	Warp Direction	Weft Direction	
Control	25.1 ± 0.7 ^c^	41.4 ± 0.4 ^c^	—
OP10	40.7 ± 0.7 ^b^	60.2 ± 1.0 ^b^	25.53 ± 2.42 ^a^
OP10-SC	44.7 ± 0.8 ^a^	58.3 ± 1.5 ^b^	26.43 ± 1.03 ^a^
OP10-SC-Si2	41.3 ± 1.4 ^b^	62.6 ± 1.2 ^a^	27.34 ± 1.70 ^a^

Data are expressed as mean ± SD (*n* = 5 for bending length; *n* = 3 for add-on). Different superscript letters within the same column indicate statistically significant differences (one-way ANOVA followed by Tukey’s HSD test (*p* < 0.05).

**Table 9:** UL 94 VBT results of PET fabrics treated with 10% *w*/*v* P-FR, 1% *w*/*v* SC, and 2% *w*/*v* Si-FR (OP10-SC-Si2) after 0–50 washing cycles.

UL 94 VBT Criteria	Number of Washing Cycles					
	0	5	10	20	30	50
T1 + T2 (s)	20.1	41	73.5	88.6	116.3	131.4
T2 + T3 ≤ 30 s	Yes	Yes	Yes	Yes	Yes	Yes
T1, T2 ≤ 10 s	No	No	No	No	No	No
T1, T2 ≤ 30 s	Yes	Yes	Yes	Yes	Yes	Yes
Afterflame/afterglow ≤ 125 mm	Yes	Yes	Yes	Yes	Yes	Yes
Dripping	Observed	Observed	Observed	Observed	Observed	Observed
Ignition of cotton	No	Yes	Yes	Yes	Yes	Yes
Classification	V-0	V-2	V-2	V-2	V-2	V-2

**Table 10:** LOI values of the fabric samples before washing and after 5 washing cycles.

Sample	LOI (%)	
	Before Washing	After Washing
Control	21.1 ± 0.4 ^b^	N/A
OP10	32.2 ± 1.1 ^a^	28.6 ± 0.5 ^b^
OP10-SC	31.3 ± 0.2 ^a^	27.0 ± 0.9 ^c^
OP10-SC-Si2	31.7 ± 0.2 ^a^	30.5 ± 0.3 ^a^

Data are expressed as mean ± SD (*n* = 5). Different superscript letters within the same column indicate statistically significant differences (one-way ANOVA followed by Tukey’s HSD test (*p* < 0.05).

**Table 11:** Comparison of phosphinate-type flame-retardant systems for PET materials reported in the literature and the present study.

Flame-Retardant System	Substrate	Processing Method	FR Loading/ Add-On	Vertical Flammability Test Results	Melt- Dripping	LOI (%)	Reference
ADP + phenyl/vinyl polysilsesquioxane (VPPSQ)	PET (bulk)	Twin-screw melt compounding + injection molding	10 wt%	V-0 (UL 94)	No	32.8	[16]
Zinc phosphinate (Exolit OP950)	PET fiber	Twin-screw extrusion (masterbatch) + melt spinning	1.4 wt%	V-2 (UL 94)	Reduced but present	24.5	[36]
Aluminium phosphinate (Exolit OP1230) + OctaMethyl POSS + DodecaPhenyl POSS)	PET fabric	Melt blending + melt spinning + knitting	10 wt%	V-2 (UL 94)	Reduced but present	Not reported	[37]
Melamine polyphosphate + ADP + carbon black/ polyacrylate adhesive	PET/spandex fabric	Padding	21.1 wt%	Damaged length: 7.1 cm; after-flame time: 0 s (GB/T 5455)	No	27.5 (before washing) 25.2 (after five washing cycles)	[38]
Aluminum 2-carboxyethyl-phenyl-phosphinate/PU coating	PET fabric	Back coating	14.7 wt%	B1 (GB/T 5455)	No	24.5	[39]
ADP + polyacrylate based on melamine–phytic acid (MP)	PET fabric	Surface coating	Not reported	B1	No	26.3	[40]
ADP (Exolit OP 1400) + SC + trisilanol isobutyl-POSS	PET fabric	Surface coating	27.34 wt%	V-0 (UL 94) (before washing) V-2 (UL 94) (after 5 washing cycles)	No (before washing) Yes (after washing)	31.7 (before washing) 30.5 (after washing)	Present study

## Data Availability

Dataset available upon request from the authors.
